# Design, Synthesis, Crystal Structure, In Vitro and In Silico Evaluation of New *N′*-Benzylidene-4-tert-butylbenzohydrazide Derivatives as Potent Urease Inhibitors

**DOI:** 10.3390/molecules27206906

**Published:** 2022-10-14

**Authors:** Sajjad Ahmad, Momin Khan, Najeeb Ur Rehman, Muhammad Ikram, Sadia Rehman, Mahboob Ali, Jalal Uddin, Ajmal Khan, Aftab Alam, Ahmed Al-Harrasi

**Affiliations:** 1 Department of Chemistry, Abdul Wali Khan University, Mardan 23200, Pakistan; 2 Natural & Medical Sciences Research Center, University of Nizwa, Nizwa 616, Oman; 3 Department of Chemistry, Government Degree College Takht Bhai, Mardan 23200, Pakistan; 4 Department of Pharmaceutical Chemistry, College of Pharmacy, King Khalid University, Abha 62529, Saudi Arabia; 5 Department of Chemistry, University of Malakand, Chakdara, Dir (L), Chakdara 18800, Pakistan

**Keywords:** synthesis, urease inhibition, 4-(tert-butyl)benzohydrazide, crystal structure, in vitro, SAR, molecular docking

## Abstract

Background: Hydrazides play a vital role in making biologically active compounds in various fields of chemistry. These determine antioxidant, antidepressant, antimalarial, anti-inflammatory, antiglycating, and antimicrobial activity. In the present study, twenty-three new *N′* benzylidene-4-(tert-butyl)benzohydrazide derivatives (**4**–**26**) were synthesized by the condensation of aromatic aldehydes and commercially available 4-(tert-butyl)benzoic acid. All the target compounds were successfully synthesized from good to excellent yield; all synthesized derivatives were characterized via spectroscopic techniques such as HREI-MS and ^1^H-NMR. Synthesized compounds were evaluated for in vitro urease inhibition. All synthesized derivatives demonstrated good inhibitory activities in the range of IC_50_ = 13.33 ± 0.58–251.74 ± 6.82 µM as compared with standard thiourea having IC_50_ = 21.14 ± 0.425 µM. Two compounds, **6** and **25**, were found to be more active than standard. SAR revealed that electron donating groups in phenyl ring have more influence on enzyme inhibition. However, to gain insight into the participation of different substituents in synthesized derivatives on the binding interactions with urease enzyme, in silico (computer simulation) molecular modeling analysis was carried out.

## 1. Introduction 

Urease (EC 3.5.1.5) belongs to the family of amidohydrolase enzymes that possesses two nickel atoms in its core structure. The conversion of urea into ammonia and carbamate is catalyzed by the action of this enzyme. The excessive amount of ammonia released due to the hyperactivity of the urease enzyme leads to the alkalinity of the stomach, which in turn increases the gastric mucosa permeability [1]. The nitrogen metabolism of cattle and various other animals is controlled by the action of the urease enzyme [2]. The elevated levels of these enzymes lead to several pathogenic conditions, such as it helps in the survival of some bacterial pathogens, thus leading to some severe side effects [3]. In humans, the low pH of the stomach facilitates the survival of *Helicobacter pylori* (HP) which leads to the development of gastric and peptic ulcers, which may eventually cause cancer [4]. The increased level of ammonia is also responsible for several metabolic disorders and destroys the GIT epithelium. A number of compounds belonging to different classes are reported as urease inhibitors, such as thiolates that bind with the nickel atom of the enzyme. Hydroxamic acid and its derivatives act as competitive inhibitors and compete with urea, phosphorodiamidates, and a few peptide chains having a ligand that may chelate with nickel of urease. Unfortunately, these molecules have adverse side effects associated with them. Therefore, it is of crucial importance to identify more urease inhibitors having significant stability, bioavailability, and low toxicity [5]. 

Day by day, the chemistry of carbon–nitrogen double bond of hydrazone is fastly becoming the backbone of condensation reaction in benzo-fused *N*-heterocycles. Hydrazides are an important class of functional groups in organic chemistry possessing -NH-N=CH- groups with the availability of proton that aids in their pharmaceutical importance. The remedial possibilities of acid hydrazides gained momentum after the innovation of isonicotinic acid hydrazide (INH). The remarkable clinical value of INH [6,7] stimulated the study of other heterocyclic hydrazides possessing mono-cyclic nuclei such as furan, pyrrole, thiophene, and dicyclic nuclei such as quinoline and isoquinoline.

Benzohydrazides are reported to possess a wide variety of biological activities such as antiglycation [8,9], antioxidant [10,11,12], antileishmanial [13], antibacterial [14], antifungal [15], antitumor [16], and anticonvulsant [17]. Benzothiazole Schiff bases have also been reported with various biological activities [18,19,20]. In search of potentially active compounds, we synthesized *N′*-benzylidene-4-(tert-butyl)benzohydrazide derivatives (**4**–**26**) and evaluated their in vitro urease inhibitory activity, and found good results. Further modifications and studies on these compounds might be helpful in bringing a number of leading compounds that may serve as potential drug candidates.

## 2. Result and Discussion

### 2.1. Chemistry

**Scheme 1:**
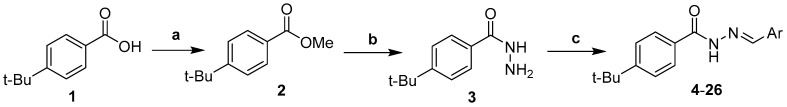
General scheme for the synthesis of *N′*-benzylidene-4-(t-Bu)benzohydrazide derivatives. (**a**) MeOH, H_2_SO_4_, reflux, 2 h, (**b**) NH_2_NH_2_, H_2_O, MeOH, reflux, 3–4 h, (**c**) Aromatic aldehydes, AcOH, MeOH, reflux, 4–6 h.

Twenty-three different *N′*-benzylidene-4-(t-Bu)benzohydrazide derivatives, (**4**–**26**) were synthesized by a routes outlined in Scheme 1. In the first step, methyl 4-tert-butylbenzoate (**2)** was synthesized from 4-(t-Bu)benzoic acid (**1)** by refluxing in methanol for two hours in the presence of concentrated H_2_SO_4_. Methyl 4-tert-butylbenzoate (**2**) was then reacted with hydrazine hydrate in methanol to give the corresponding hydrazide **3**. The diversely substituted hydrazones (**4**–**26**) were obtained by refluxing different substituted aromatic aldehydes with 4-(tBu)benzohydrazide in the presence of methanol and glacial acetic acid with continuous stirring to get *N′*-benzylidene-4-(tert-butyl)benzohydrazide derivatives **4**–**26**. TLC technique was used to monitor the reaction progress periodically. For the confirmation of newly synthesized benzohydrazide derivatives **4**–**26** (Table 1) [21], spectroscopic techniques such as ^1^H-, ^13^C-NMR, and HREI-MS were performed.

### 2.2. Crystal Structure Description

The crystal structure of 4-tert-butyl-*N*′-[(E)-(4-fluoro-3-methoxyphenyl)methylidene]benzohydrazide (**4+**) has been reported previously [22].

#### 2.2.1. Crystal Structure of 4-(tBu)-*N*′-(2-Hydroxy-3-methoxybenzylidene)benzohydrazide (**5**)

**Figure 1:**
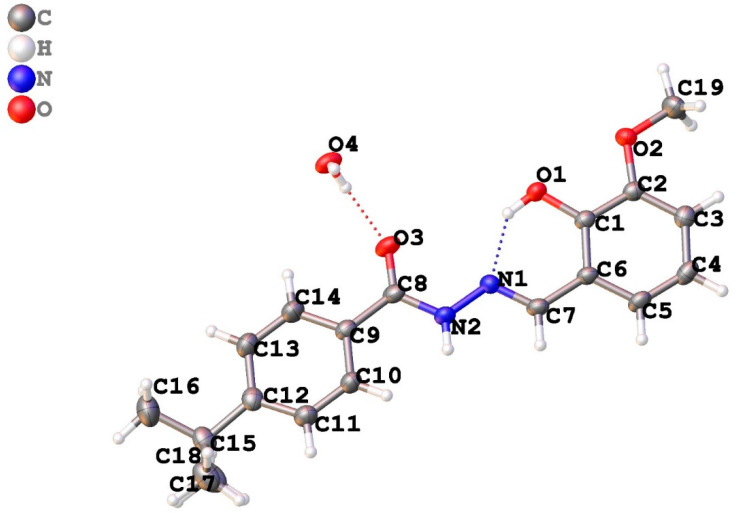
Molecular structure of 4-(t-Bu)-*N′*-(2-hydroxy-3-methoxybenzylidene)benzohydrazide (**5**).

The 4-(tBu)-*N′*-(2-hydroxy-3-methoxybenzylidene)benzohydrazide (**5**) crystallized in the monoclinic, *Cc* space group. The asymmetric unit of **5** is shown in Figure 1. The crystal data of **5** is shown in Table 2, whereas the selected bond lengths and angles are shown in Table 3. The O3-C8 bond length is negligibly shorter than a similar bond length in compound **14**. Similarly, the N-N bond length in compound **5** is 1.364 Å, comparably shorter than a similar bond length in **14**. The reason coined for this effect is the substitution of hydroxyl and methoxy groups at *ortho* and *meta* (C1 and C2) positions of the benzene ring. Similar behavior is observed in bond lengths as well. Therefore, we will restrict the crystal description to **14** only.

**Figure 2:**
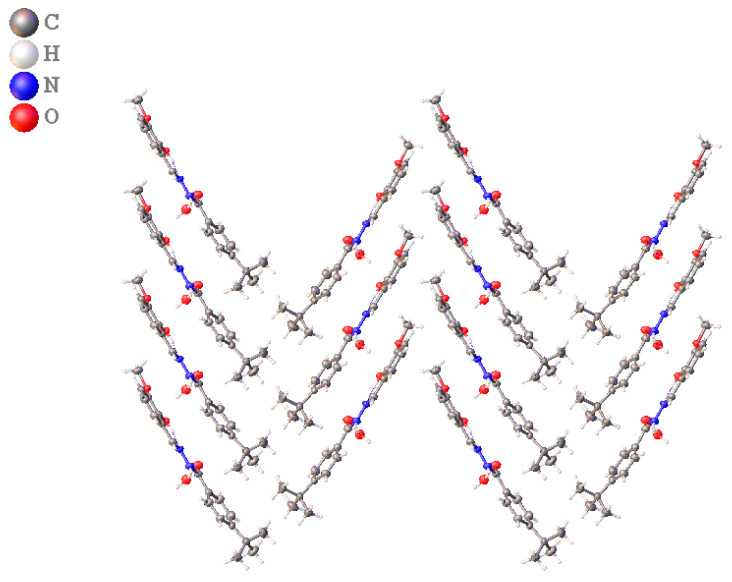
Crystal packing diagram of 4-(t-Bu)-*N′*-(2-hydroxy-3-methoxybenzylidene)benzohydrazide (**5**).

The hydrogen bonding is unique in **5,** as shown in Table 4. Overall, there is a single intramolecular hydrogen bonding interaction shown by O1—H1*O*···N1 with a length of 2.581 (**4**) Å showing strong interaction. Similarly, there are intermolecular hydrogen bonds as well, which lead to the accumulation of a co-crystallized water molecule in the crystal lattice. The hydrogen bonding interaction is shown in Table 4. The crystal packing diagram of **5** is shown in Figure 2.

#### 2.2.2. Crystal Structure of 4-Tert-butyl-*N*′-{(*E*)-[4-(dimethylamino)phenyl]methylidene}benzohydrazide (**14**)

**Figure 3:**
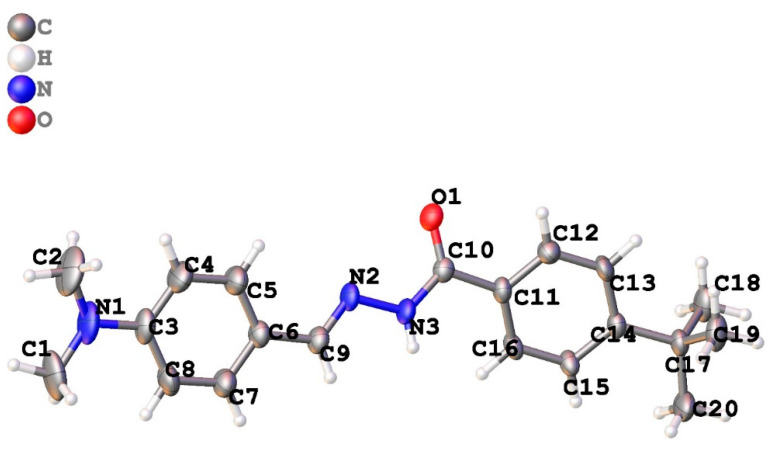
Molecular Structure of compound **14**.

**Figure 4:**
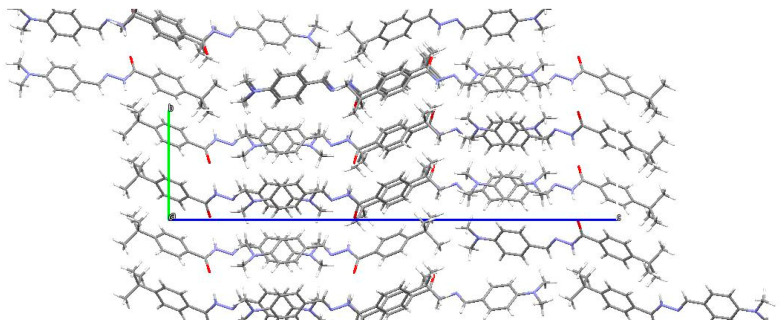
Crystal packing diagram of compound **14**.

The resulting hydrazone was crystallized in a monoclinic *Pbca* space group with only one molecule in the asymmetric unit, as shown in Figure 3. The bond lengths and bond angles of the molecule fall within the expected range for similar molecules [22,23,24,25,26,27,28,29,30,31,32]. The crystal data for the compound is given in Table 2, whereas the bond lengths and the reported ranges are given in Table 5. It is apparent from the table that the bond length for the hydrazide moiety N2–N3 is 1.395 (3) Å, for the Schiff base linkage N2–C9 is 1.260 (**4**) Å, and for the N3–C10 is 1.332 (**4**) [24,25,26,27,28,29,30,31,32,33,34]. The two phenyl rings connected by hydrazide moiety are rather twisted in comparison with other related examples. The twist is identified from the 15.46 torsion between the two phenyl planes. The value is lying on the coplanar side of the range (4.74°) reported earlier for similar compounds. The connecting hydrazide moiety along the Schiff base imine group (O1–C10–N3–N2–C9) produces a plane lying at the 11.13° and 23.21° to the planes produced by C5–C4–C3–C8–C7–C6 and C14–C13–C12–C11–C16–C15, respectively. The torsion in planes is apparently influenced by the crystal packing effects and hydrogen bonding. The crystal lattice of the hydrazide is marked by the strong hydrogen bonding (Table 6) of D···A = 2.939 (3)Å {N3—H1N3···O1i} and D···A = 3.322 (4) Å { C9—H9···O1i}. The crystal packing diagram of the compound is shown in Figure 4.

### 2.3. In Vitro Urease Inhibitory Activity

In vitro urease, inhibition was detected for the synthesized *N*′-benzylidene-4-(t-Bu) benzohydrazide derivatives (**4**–**26**), which indicated inhibition in the range of IC_50_ = 13.33 ± 0.58 µM to IC_50_ = 251.74 ± 6.82 µM as compared with standard thiourea IC_50_ = 21.14 ± 0.425 µM (Table 7).

### 2.4. Structure-Activity Relationship (SAR)

**Figure 5:**
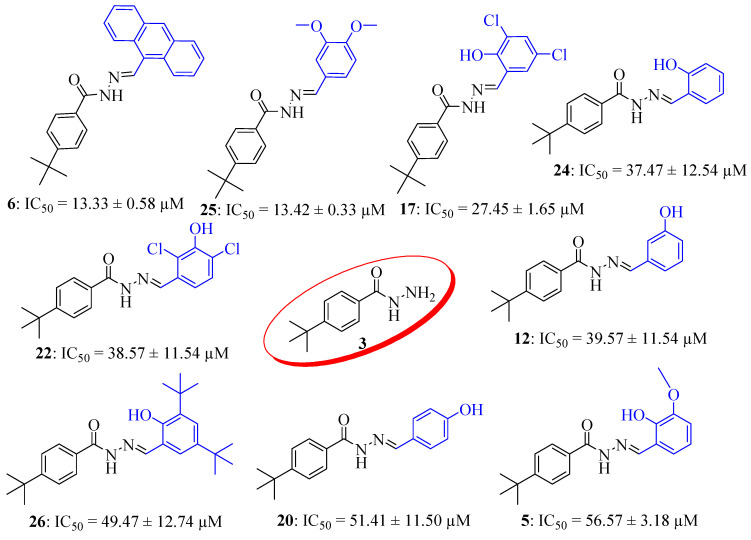
Active inhibitors of the synthesized series.

To explain the structure–activity relationship (SAR) of these analogs, we have classified the top eight compounds of the series into two groups according to their nature of substituents. Limited SAR study was established for synthesized derivatives **4**–**26**. The most active compounds among the series were compounds **6** and **25,** with IC_50_ values of 13.33 ± 0.58 and 13.42 ± 0.33 µM when compared with standard thiourea (IC_50_ = 21.14 ± 0.425 µM) (Table 7). The potency of compounds **6** and **25** might be due to the presence of anthracene and di-methoxy moiety, respectively. In both cases, the substituents are electron rich in nature. Compounds **17**, **24**, **22**, **12**, **26**, **20**, and **5** are the second group of active compounds having hydroxyl groups as substituents. The activity of these analogs might be due to the hydroxyl group (Figure 5). It seems that the remaining compounds showed much lower activity than the standard. Results of the enzyme inhibition showed that the nature of the substituents played an important role in enhancing the inhibition potential of the core structure. Apart from it, both compounds **5** and **14** studied crystallographically also reveal activity. Compound **5** with IC_50_ = 56.57 ± 3.18 µM is revealing better activity than the compound **14** (IC_50_ = 81.21 ± 7.4 µM). The possible explanation for this behavior may be linked to the presence and absence of methoxy moiety. Compound **5** revealed almost identical activity to other similar analogs such as **20**. Both the derivatives possess ortho-substituted hydroxyl group, which is involved in hydrogen bonding with hydrazide moiety, as may be seen in the crystal structure of compound **5**.

### 2.5. Molecular Docking, Interactions Report

To predict the inhibition mechanism of isolated compounds shown by the kinetics study, molecular docking of the active compounds was carried out with the crystal structure of the urease enzyme. The most favorable docking conformations of all compounds were observed inside the active site with proper orientation. The active site consists of both the hydrophobic and hydrophilic amino acids.

The hydrophilic amino acids included Glu166, 223, Arg339, His323, 324, Asp224, 363, and Asp 494, while the hydrophobic part was composed of Lys169, Ala170, 366, Leu319, Cys322, and Met 637. The two Ni ions also played a vital role by linking the key amino acid and ligands. It has been observed that almost all the conformations of all the ligands showed interactions with key residues inside the pocket. The docked poses were ranked by the scores from the GBVI/WSA binding free energy calculation. The most promising docked conformation of each compound was further evaluated for binding mode. Detailed docking results are listed in Table 8.

**Figure 6:**
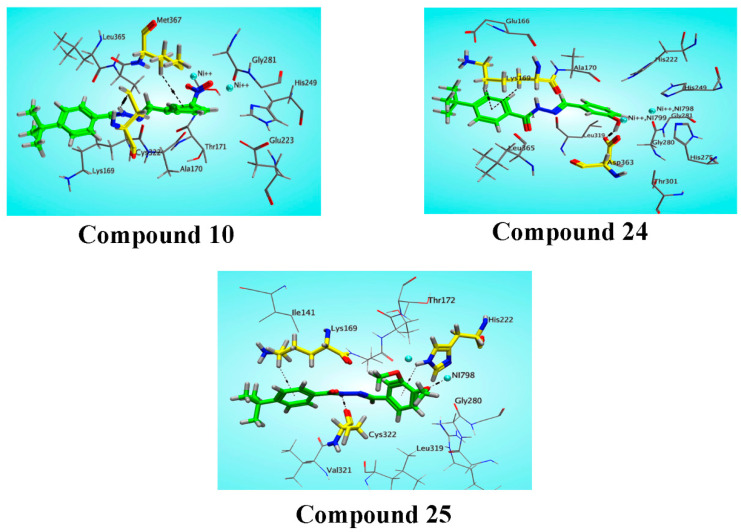
Three dimensional interaction of compounds **10**, **24**, **25**, with active site residues of urease. Ligands are shown in green, key residues of the active site are shown in yellow stick mode, hydrogen bonding and other interaction is shown in dark color dotted lines.

The binding mode of the active compounds with the active site residues showed a healthy assignation with the backbone of the enzyme using hydrogen bonds, polar bonds, pi-pi, and pi-H interactions. Three-dimensional interactions of some favorable inhibitors are shown in Figure 6. This showed that ligands occupy the active site residues, using hydrogen bonds, polar bonds, arene-cation, and metal ion interactions to engage the backbone of the enzyme tightly.

In the case of compound **10**, the chloronitrotoluene part, NO_2_ and NH are found actively interacting with the active site residues Cys 322, Met 367, and Ni 798 through Hydrogen bonding, π-Hydrogen, and metal ion interactions, respectively. The phenolic substituted derivative **24** is observed with lesser interactions compared with the chloro-nitrobenzene derivative **10** by performing two interactions with Lys 169 and Asp 363. Asp 363 established a Hydrogen donor interaction with the OH group of the ligand while Lys 169 formed the H-π interaction with the aromatic π system of the ligand. The hydrazine moiety presented an inert behavior that might be attributed to the negative inductive effect of these groups.

The dimethoxy substituted derivative, compound **25**, showed better interactions and docking scores in accordance with the biological activity. Figure 5 reflects that hydrazine moiety in compound **25** performed Hydrogen bonding with Cys 322, and the substituted methoxy group established metal/ionic contact with Ni 798 of the target protein. Moreover, the two aromatic rings pi system interact through π-H bonding to Lys 169 and His 222.

The docking study complemented the experimental results based on the multiple interactions of ligands with key residues of the urease enzyme. The docking pose of the most active compounds, **10**, **24**, and **25**, inhibited the catalytic activities of the urease by binding firmly through strong hydrogen bonding, hydrophobic, polar interactions with key residues, and metal/ion contact.

The molecular docking study of these compounds revealed that the ligands with polar, light, and electron-rich groups such as hydroxyl, alkoxy, and nitro groups showed better interaction mode and high docking scores against the target protein and therefore had good inhibitory activities. On the other hand, ligands with electron-withdrawing groups, non-polar such as methyl or benzene, and bulky groups have shown poor interactions and low docking scores.

## 3. Experimental

### 3.1. Materials and Methods

NMR experiments were performed on Avance-Bruker (400.15 MHz for ^1^H). The mass spectra were recorded by HRESI-MS spectrometer (LCQ-DECA XP Plus, Thermo-Finnigan, San Diego, CA, USA). Thin layer chromatography (TLC) was performed on pre-coated silica gel aluminum plates (Kieselgel 60, 254, E. Merck, Darmstadt, Germany). Chromatograms were visualized by UV visible light at 254 nm or iodine vapors.

Highly pure analytical grade chemicals and solvents were procured from (Darmstadt, Germany) and used as received. Aryl benzaldehyde derivatives and 4-(t-Bu)benzoic acid were purchased from Sigma (St Louis, MO, USA). Sodium hydroxide, soluble starch, maltose, and other chemicals were obtained from Merck (Darmstadt, Germany).

#### 3.1.1. General Procedure for the Synthesis of Methyl-4-(t-Bu)benzoate (**2**) and 4-(Tert-butyl) benzohydrazide (**3**)

4-(tert-butyl)benzoic acid **1** (2.5 g, 0.014 mmol) was suspended in methanol (25 mL), and concentrated sulphuric acid (H_2_SO_4_) (1.2 mL) was added to the flask, and refluxed for 2 h. Upon completion, the reaction mixture was poured into a beaker containing ice-cold distilled water and neutralized the mixture with sodium bicarbonate. Extracted with organic solvent (chloroform) to obtain 4-(tert-butyl)benzoic acid **2**. It was then recrystallized with methanol. Methyl 4-tert-butylbenzoate **2** was refluxed with hydrazine hydrate (1.5 eqv.) in methanol (10 mL) for 3–4 h to obtain the 4-tert-butylbenzohydrazide **3**. After the reaction, precipitates were formed, filtered, washed with distilled water, dried under a vacuum, and collected.

#### 3.1.2. General Procedure for the Synthesis of *N*-Acylhydrazones 4-(t-But)benzohydrazide (**4**–**26**)

Twenty-three different novel hydrazones (**4**–**26**) of 4-(t-But)benzohydrazide **3** were synthesized in good to excellent yields. In a typical reaction, 4-(tert-butyl)benzohydrazide **3** (0.192 g, 0.001 mmol) was dissolved in methanol (10 mL) containing a catalytic amount of acetic acid, then various substituted aromatic aldehydes (0.001 mmol) were added to the flask and refluxed it for 4–6 h. The reaction mixture was decanted into a beaker containing crushed ice. Precipitates were formed, filtered, and washed with an excess of water and hot *n*-hexane, and the products were dried and collected [21]. All the synthesized derivatives were characterized by HRESI-MS and ^1^H-NMR spectroscopy. Furthermore, we have already reported the crystal structure of compound **4** [22].

### 3.2. Urease Inhibition Assay

The measurement of urease inhibitory activity was carried out according to the literature method [33]. The assay mixture containing 75 μL of Jack bean urease and 75 μL of tested compounds with various concentrations (dissolved in DMSO) was pre-incubated for 15 min on a 96-well assay plate. Acetohydroxamic acid was used as a reference. Then 75 μL of phosphate buffer at pH 6.8 containing phenol red (0.18 mmol·L^−1^) and urea (400 mmol·L^−1^) were added and incubated at room temperature. The reaction time required for enough ammonium carbonate to form to raise the pH phosphate buffer from 6.8 to 7.7 was measured by a microplate reader (560 nm), with the end-point being determined by the color change of the phenol-red indicator.

### 3.3. Docking Methodology

A molecular docking study was performed according to the reported methodology [34] to predict the binding mode of the active synthesized compounds in the active site of the urease enzyme using MOE (Molecular Operating Environment). The three-dimensional structures of the isolated compounds were built using the builder tool implemented in MOE software. The generated compounds were 3D protonated, and energy minimized using the default parameters of the MOE (gradient: 0.05, Force Field: MMFF94X). 3D structure of the target protein was retrieved from the protein databank (https://www.rcsb.org/ accessed on 4 August 2022) (PDB ID 4ubp), the solvent molecules were removed, and 3D protonation was carried out. To get a stable conformation of the protein molecule, 3D protonation of the protein was energy minimized using the default parameters of MOE. For docking studies, the parameters of MOE used were Placement: Triangle Matcher, Rescoring 1: London dG, Refinement: Forcefield, Rescoring 2: GBVI/WSA. For each ligand, 10 conformations were allowed to be formed, and the top-ranked conformations on the basis of docking score were selected for further analysis.

### 3.4. Crystal Structure Determination

Crystals **1**, **5**, and **14** were mounted on a glass fiber in inert paraffin oil. Data were recorded at 170 K on an STOE-IPDS 2T diffractometer with graphite-monochromated Mo-K_α_-radiation (λ = 0.71073 Å). The program XArea was used to integrate diffraction profiles; numerical absorption corrections were carried out with the programs X-Shape and X-Red32, all from STOE© version 2010. The structures were solved by dual space methods (SHELXT-2016) [35] and refined by full-matrix least-squares techniques using the WingX GUI [36] and SHELXL-2018 [37]. All non-hydrogen atoms were refined with anisotropic displacement parameters. The hydrogen atoms were refined isotropically on calculated positions using a riding model with their *U*_iso_ values constrained to 1.5 *U*_eq_ of their pivot atoms for terminal sp^3^ carbon atoms and 1.2 times for all other carbon atoms.

Crystallographic data were deposited with the Cambridge Crystallographic Data Centre, CCDC, 12 Union Road, Cambridge CB21EZ, UK. These data can be obtained free of charge by quoting the depository numbers CCDC 2166390 (compound **5**) and 1861240 (compound **14**) by FAX (+44-1223-336-033), email (deposit@ccdc.cam.ac.uk) or their web interface (http://www.ccdc.cam.ac.uk accessed on 4 August 2022). 

### 3.5. Analytical Physical and Spectroscopic Data of the Synthesized Compounds

#### 3.5.1. 4-(Tert-butyl)-*N*′-(4-fluoro-3-methoxybenzylidene)benzohydrazide (**4**)

Yield: (72%), Crystal; *Rf*: 0.51 (*n*-hexane/ethyl acetate (2.5:7.5)); ^1^H NMR (400 MHz DMSO-d_6_) *δ* 1.39 (s, 9H), 3.89 (s, 3H), 6.98 (d, *J* = 7.5 Hz, 1H), 7.27 (d, *J* = 7.5 Hz, 1H), 7.47 (d, *J* = 7.0 Hz, 2H), 7.87 (d, *J* = 7.0 Hz, 2H), 8.12 (m, 1H), 8.58 (s, 1H), 11.68 (s, 1H); HRESI-MS [M + H]^+^ calculated for C_19_H_21_FN_2_O_2_, 328.1587, found 328.1598.

#### 3.5.2. 4-(t-But)-*N*′-(2-Hydroxy-3-methoxybenzylidene)benzohydrazide (**5**)

Yield: (82%), Cream color; *Rf*: 0.52 (*n*-hexane/ethyl acetate (2.0:8.0)); ^1^HNMR (400 MHz DMSO-d_6_) *δ* 1.21 (s, 9H), 3.84 (s, 3H), 6.96 (d, *J* = 7.2 Hz, 1H), 7.19 (t, *J* = 7.2 Hz, 1H), 7.25 (d, *J* = 7.2 Hz, 1H), 7.43 (d, *J* = 7.0 Hz, 2H), 7.82 (d, *J* = 7.0 Hz, 2H), 8.53 (s, 1H), 10.55 (br.s, 1H), 11.58 (s, 1H); HRESI-MS [M + H]^+^ calculated for C_19_H_22_N_2_O_3_, 326.1630, found 326.1643.

#### 3.5.3. *N*′-(Anthracen-9-ylmethylene)-4-(tert-butyl)benzohydrazide (**6**)

Yield: (78%), Color: Light blue; *Rf*: 0.50 (*n*-hexane/ethyl acetate (2.5:7.5)); ^1^HNMR (400 MHz DMSO-d_6_) *δ* 1.28 (s, 9H), 7.34 (t, *J* = 7.5 Hz, 4H), 7.49 (d, *J* = 7.2 Hz, 2H), 7.68–7.70 (m, 4H), 7.81 (d, *J* = 7.2 Hz, 2H), 8.07 (s, 1H), 8.53 (s, 1H), 11.61 (s, 1H); HRESI-MS [M + H]^+^ calculated for C_26_H_24_N_2_O, 380.1889, found 380.1897.

#### 3.5.4. *N*′-(5-Bromo-2-methoxybenzylidene)-4-(t-But)benzohydrazide (**7**)

Yield: (85%), Light brown color; *Rf*: 0.50 (*n*-hexane/ethyl acetate (2.5:7.5)); ^1^HNMR (400 MHz DMSO-d_6_) *δ* 1.25 (s, 9H), 3.81 (s, 3H), 6.89 (d, *J* = 7.0 Hz, 1H), 7.24 (d, *J* = 7.0 Hz, 1H), 7.41 (d, *J* = 7.5 Hz, 2H), 7.84 (d, *J* = 7.5 Hz, 2H), 7.96 (s, 1H), 8.50 (s, 1H), 11.54 (s, 1H); HRESI-MS [M + H]^+^ calculated for C_19_H_21_BrN_2_O_2_, 388.0786 found 388.0793.

#### 3.5.5. *N*′-(3,4-Dihydroxybenzylidene)-4-tert-butylbenzohydrazide (**8**)

Yield: (70%), Cream color; *Rf*: 0.50 (*n*-hexane/ethyl acetate (2.0:8.0)); ^1^HNMR (400 MHz DMSO-d_6_) *δ* 1.26 (s, 9H), 6.80 (d, *J* = 7.1 Hz, 1H), 7.11 (d, *J* = 7.1 Hz, 1H), 7.48 (d, *J* = 7.5 Hz, 2H), 7.89 (d, *J* = 7.5 Hz, 2H), 8.48 (s, 1H), 10.54 (br.s, 1H), 10.82 (br.s, 1H), 11.62 (s, 1H); HRESI-MS [M + H]^+^ calculated for C_18_H_20_N_2_O_3_, 312.1474 found 312.1490.

#### 3.5.6. *N*′-(2,3,4-Trimethoxybenzylidene)-4-tert-butylbenzohydrazide (**9**)

Yield: (75%), Cream color; *Rf*: 0.54 (*n*-hexane/ethyl acetate (2.5:7.5)); ^1^H NMR (400 MHz DMSO-d_6_) *δ* 1.39 (s, 9H), 3.63 (s, 9H), 6.85 (d, *J* = 7.0 Hz, 1H), 7.17 (d, *J* = 7.0 Hz, 1H), 7.44 (d, *J* = 7.0 Hz, 2H), 7.81 (d, *J* = 7.0 Hz, 2H), 8.50 (s, 1H), 11.68 (s, 1H); HRESI-MS [M + H]^+^ calculated for C_21_H_26_N_2_O_4_, 370.1942, found 370.1967.

#### 3.5.7. 4-(Tert-butyl)-*N*′-(2-chloro-5-nitrobenzylidene)benzohydrazide (**10**)

Yield: (88%), Cream color; *Rf*: 0.50 (*n*-hexane/ethyl acetate (2.5:7.5)); ^1^H NMR (400 MHz DMSO-d_6_) *δ* 1.26 (s, 9H), 7.48 (d, *J* = 7.5 Hz, 2H), 7.58 (d, *J* = 7.5 Hz, 2H), 7.86 (d, *J* = 9.0 Hz, 1H), 8.48 (s, 1H), 8.61 (d, *J* = 9.0 Hz, 1H), 8.74 (d, *J* = 1.5 Hz, 1H), 11.62 (s, 1H); HRESI-MS [M + H]^+^ calculated for C_18_H_18_ClN_3_O_3_, 359.1037, found 359.1087.

#### 3.5.8. 4-(Tert-butyl)-*N*′-(4-ethoxy-2-methoxybenzylidene)benzohydrazide (**11**)

Yield: (78%), Cream color; (*n*-hexane/ethyl acetate (2.5:7.5)); *Rf*: 0.53 (N-Hexane/Ethylacetate 25:75); ^1^HNMR (400 MHz DMSO-d_6_) *δ* 1.41 (s, 9H), 2.01 (t, *J* = 6.8 Hz, 3H), 3.86 (q, *J* = 6.8 Hz, 2H), 3.89 (s, 3H), 6.68 (d, *J* = 1.0 Hz, 1H), 6.97 (d, *J* = 7.0 Hz, 1H), 7.28 (d, *J* = 7.0 Hz, 1H), 7.44 (d, *J* = 7.5 Hz, 2H), 7.87 (d, *J* = 7.5 Hz, 2H), 8.45 (s, 1H), 11.63 (s, 1H); HRESI-MS [M + H]^+^ calculated for C_21_H_26_N_2_O_3_, 354.1943, found 354.1952.

#### 3.5.9. 4-(Tert-butyl)-*N*′-(3-hydroxybenzylidene)benzohydrazide (**12**)

Yield: (88%), Light brownish color; *Rf*: 0.52 (*n*-hexane/ethyl acetate (2.5:7.5)); ^1^HNMR (400 MHz DMSO-d_6_) *δ* 1.38 (s, 9H), 6.68 (d, *J* = 1.0 Hz, 1H), 6.92 (d, *J* = 7.2 Hz, 1H), 6.99 (t, *J* = 7.2 Hz, 1H), 7.18 (d, *J* = 7.2 Hz, 1H), 7.42 (d, *J* = 8.0 Hz, 2H), 7.88 (d, *J* = 8.0 Hz, 2H), 8.41 (s, 1H), 10.57 (s, 1H), 11.59 (s, 1H); HRESI-MS [M + H]^+^ calculated for C_18_H_20_N_2_O_2_, 296.1525, found 296.1576.

#### 3.5.10. 4-(Tert-butyl)-*N*′-(3,4,5-trimethoxybenzylidene)benzohydrazide (**13**)

Yield: (80%), Light yellow color; *Rf*: 049 (*n*-hexane/ethyl acetate (2.0:8.0)); ^1^HNMR (400 MHz DMSO-d_6_) *δ* 1.31 (s, 9H), 3.76 (s, 9H), 6.62 (d, *J* = 1.5 Hz, 2H), 7.40 (d, *J* = 7.5 Hz, 2H), 7.83 (d, *J* = 7.5 Hz, 2H), 8.48 (s, 1H), 11.64 (s, 1H); HRESI-MS [M + H]^+^ calculated for C_21_H_26_N_2_O_4_, 370.1893, found 370.1923.

#### 3.5.11. 4-(Tert-butyl)-*N*′-(4-(dimethylamino)benzylidene)benzohydrazide (**14**)

Yield: (85%), Cream color; *Rf*: 0.50 (*n*-hexane/ethyl acetate (2.0:8.0)); ^1^HNMR (400 MHz DMSO-d_6_) *δ* 1.34 (s, 9H), 2.76 (s, 6H), 6.94 (d, *J* = 7.0 Hz, 2H), 7.38 (d, *J* = 7.6 Hz, 2H), 7.52 (d, *J* = 7.6 Hz, 2H), 7.89 (d, *J* = 7.0 Hz, 2H), 8.38 (s, 1H), 11.36 (s, 1H); HRESI-MS [M + H]^+^ calculated for C_20_H_25_N_3_O, 323.1998, found 323.2022.

#### 3.5.12. 4-(Tert-butyl)-*N*′-(2,4-dichlorobenzylidene)benzohydrazide (**15**)

Yield: (90%), Cream color; *Rf*: 0.54 (*n*-hexane/ethyl acetate (2.5:7.5)); ^1^HNMR (400 MHz DMSO-d_6_) *δ* 1.42 (s, 9H), 6.68 (d, *J* = 1.0 Hz, 1H), 7.12 (d, *J* = 7.1 Hz, 1H), 7.25 (d, *J* = 1.2 Hz, 1H), 7.41 (d, *J* = 7.5 Hz, 2H), 7.57 (d, *J* = 7.1 Hz, 1H), 7.86 (d, *J* = 7.5 Hz, 2H), 8.39 (s, 1H), 11.53 (s, 1H); HRESI-MS [M + H]^+^ calculated for C_18_H_18_Cl_2_N_2_O, 348.0796, found 348.0875.

#### 3.5.13. 4-(Tert-butyl)-*N*′-(3,5-di-tert-butyl-4-hydroxybenzylidene)benzohydrazide (**16**)

Yield: (78%), Cream color; *Rf*: 0.52 (*n*-hexane/ethyl acetate (2.5:7.5)); ^1^HNMR (400 MHz DMSO-d_6_) *δ* 1.37 (s, 9H), 1.38 (s, 18H), 7.21 (d, *J* = 1.5 Hz, 2H), 7.47 (d, *J* = 7.5 Hz, 2H), 7.82 (d, *J* = 7.5 Hz, 2H), 8.54 (s, 1H), 10.62 (s, 1H), 11.55 (s, 1H); HRESI-MS [M + H]^+^ calculated for C_26_H_36_N_2_O_2_, 408.2777, found 408.2794.

#### 3.5.14. *N*′-(3,5-Dichloro-2-hydroxybenzylidene)-4-tert-butylbenzohydrazide (**17**)

Yield: (80%), Light yellow color; *Rf*: 0.52 (*n*-hexane/ethyl acetate (2.5:7.5)); ^1^HNMR (400 MHz DMSO-d_6_) *δ* 1.31 (s, 9H), 7.16 (d, *J* = 1.0 Hz, 1H), 7.23 (d, *J* = 1.0 Hz, 1H), 7.42 (d, *J* = 7.5 Hz, 2H), 7.88 (d, *J* = 7.5 Hz, 2H), 8.50 (s, 1H), 10.60 (s, 1H), 11.51 (s, 1H); HRESI-MS [M + H]^+^ calculated for C_18_H_18_Cl_2_N_2_O_2_, 364.1058, found 364.1069.

#### 3.5.15. *N*′-(4-Nitrobenzylidene)-4-tert-butylbenzohydrazide (**18**)

Yield: (88%), Cream color; *Rf*: 0.53 (*n*-Hexane/ethyl acetate (3.0:7.0); ^1^HNMR (400 MHz DMSO-d_6_) ) *δ* 1.34 (s, 9H), 7.44 (d, *J* = 7.5 Hz, 2H), 7.82 (d, *J* = 7.5 Hz, 2H), 8.01 (d, *J* = 9.0 Hz, 2H), 8.52 (s, 1H), 8.56 (d, *J* = 9.0 Hz, 2H), 11.39 (s, 1H); HRESI-MS [M + H]^+^ calculated for C_18_H_19_N_3_O_3_, 325.1739, found 325.1752.

#### 3.5.16. 4-(Tert-butyl)-*N*′-(2,6-dimethoxybenzylidene)benzohydrazide (**19**)

Yield: (65%), Cream color; *Rf*: 0.50 (*n*-hexane/ethyl acetate (3.0:7.0)); ^1^HNMR (400 MHz DMSO-d_6_) *δ* 1.31 (s, 9H), 3.58 (s, 6H), 6.87 (d, *J* = 6.8 Hz, 2H), 7.23 (t, *J* = 6.8 Hz, 1H), 7.48 (d, *J* = 8.0 Hz, 2H), 7.85 (d, *J* = 8.0 Hz, 2H), 8.52 (s, 1H), 11.63 (s, 1H); HRESI-MS [M + H]^+^ calculated for C_20_H_24_N_2_O_3_, 340.1787, found 340.1805.

#### 3.5.17. 4-(t-But)-*N*′-(4-Hydroxybenzylidene)benzohydrazide (**20**)

Yield: (70%), Cream color; *Rf*: 0.52 (*n*-hexane/ethyl acetate (3.0:7.0)); ^1^HNMR (400 MHz DMSO-d_6_) *δ* 1.32 (s, 9H), 6.93 (d, *J* = 7.0 Hz, 2H), 7.22 (d, *J* = 7.0 Hz, 2H), 7.45 (d, *J* = 8.0 Hz, 2H), 7.91(d, *J* = 8.0 Hz, 2H), 8.58 (s, 1H), 10.76 (s, 1H), 11.63 (s, 1H); HRESI-MS [M + H]^+^ calculated for C_18_H_20_N_2_O_2_, 296.1538, found 296.1548.

#### 3.5.18. *N*′-(2,4,6-Trimethoxybenzylidene)-4-tert-butylbenzohydrazide (**21**)

Yield: (82%), White color; *Rf*: 0.52 (*n*-hexane/ethyl acetate (2.5:7.5)); ^1^HNMR (400 MHz DMSO-d_6_) *δ* 1.35 (s, 9H), 3.63 (s, 6H), 3.71 (s, 3H), 6.68 (d, *J* = 1.0 Hz, 2H), 7.41 (d, *J* = 7.5 Hz, 2H), 7.85 (d, *J* = 7.5 Hz, 2H), 8.59 (s, 1H), 11.66 (s, 1H); HRESI-MS [M + H]^+^ calculated for C_21_H_26_N_2_O_4_, 370.1893, found 370.1923.

#### 3.5.19. 4-(Tert-butyl))-*N*′-(2,4-dichloro-3-hydroxybenzylidene)benzohydrazide (**22**)

Yield: (90%), Cream color; *Rf*: 0.51 (*n*-hexane/ethyl acetate (3.0:7.0)); ^1^HNMR (400 MHz DMSO-d_6_) *δ* 1.24 (s, 9H), 7.16 (d, *J* = 7.2 Hz, 1H), 7.29 (d, *J* = 1.5 Hz, 1H), 7.49 (d, *J* = 7.0 Hz, 2H), 7.83 (d, *J* = 7.0 Hz, 2H), 10.46 (s, 1H), 11.60 (s, 1H); HRESI-MS [M + H]^+^ calculated for C_18_H_18_C_l2_N_2_O_2_, 364.0745, found 365.0755.

#### 3.5.20. 4-(Tert-butyl))-*N*′-(3-methoxy-4-hydroxybenzylidene)benzohydrazide (**23**)

Yield: (85%), Color: Cream; *Rf*: 0.52 (*n*-hexane/ethyl acetate (3.0:7.0)); ^1^HNMR (400 MHz DMSO-d_6_) *δ* 1.35 (s, 9H), 3.75 (s, 3H), 6.88 (d, *J* = 7.2 Hz, 1H), 7.06 (s, 1H), 7.35 (d, *J* = 7.2 Hz, 1H),7.44 (d, *J* = 8.0 Hz, 2H), 7.89 (d, *J* = 8.0 Hz, 2H), 8.58 (s, 1H), 10.61 (s, 1H), 11.65 (s, 1H); HRESI-MS [M + H]^+^ calculated for C_19_H_22_N_2_O_3_, 326.1630, found 326.1646.

#### 3.5.21. 4-(t-But)-*N*′-(2-Hydroxybenzylidene)benzohydrazide (**24**)

Yield: (80%), Color: Cream; *Rf*: 0.51 (*n*-hexane/ethyl acetate (2.0:8.0)); ^1^HNMR (400 MHz DMSO-d_6_) *δ* 1.24 (s, 9H), 7.01 (d, *J* = 7.2 Hz, 1H), 7.29 (t, *J* = 7.2 Hz, 1H), 7.49 (t, *J* = 7.2 Hz, 2H), 7.58 (d, *J* = 7.2 Hz, 1H), 7.63 (d, *J* = 7.2 Hz, 2H), 7.83 (d, *J* = 7.0 Hz, 2H), 8.52 (s, 1H), 10.74 (s, 1H), 11.60 (s, 1H); HRESI-MS [M + H]^+^ calculated for C_18_H_20_N_2_O_2_, 296.1525, found 296.1535.

#### 3.5.22. 4-(t-But)-*N*′-(3,4-Dimethoxybenzylidene)benzohydrazide (**25**)

Yield: (70%), Color: Cream; *Rf*: 0.52 (*n*-hexane/ethyl acetate (2.0:8.0)); ^1^HNMR (400 MHz DMSO-d_6_) *δ* 1.35 (s, 9H), 3.75 (s, 3H), 3.76 (s, 3H), 6.88 (d, *J* = 7.2 Hz, 1H), 7.06 (s, 1H), 7.35 (d, *J* = 7.2 Hz, 1H),7.44 (d, *J* = 7.6 Hz, 2H), 7.89 (d, *J* = 7.6 Hz, 2H), 8.53 (s, 1H), 11.69 (s, 1H); HRESI-MS [M + H]^+^ calculated for C_20_H_24_N_2_O_3_, 340.1787, found 340.1802.

#### 3.5.23. 4-(t-But)-*N*′-(3,5-Di-tert-butyl-2-hydroxybenzylidene)benzohydrazide (**26**)

Yield: (90%), Color: Cream; *Rf*: 0.52 (*n*-hexane/ethyl acetate (2.5:7.5)); ^1^HNMR (400 MHz DMSO-d_6_) *δ* 1.28 (s, 9H), 1.33 (s, 9H), 1.37 (s, 9H), 7.21 (s, 1H), 7.32 (s, 1H), 7.48 (d, *J* = 8.0 Hz, 2H), 7.81 (d, *J* = 8.0 Hz, 2H), 8.50 (s, 1H), 10.58 (s, 1H), 11.69 (s, 1H); HRESI-MS [M + H]^+^ calculated for C_26_H_36_N_2_O_2_, 408.2777, found 408.2783.

## 4. Conclusions

Twenty-three (**4**–**26**) different *N*-acylhydrazones of 4-(tert-butyl)benzohydrazide were synthesized in good to excellent yields, and these compounds were structurally deduced with the help of HRESI-MS and ^1^H-NMR spectroscopy. Finally, all the derivatives were screened for their in vitro urease inhibitory potential. Eight compounds showed good to excellent inhibitory potentials for the urease enzyme. Among the series, compound **6** (IC_50_ = 13.33 ± 0.58 µM) and **25** (IC_50_ = 13.42 ± 0.33 µM) were found as lead compounds with better inhibition then the standard thiourea (IC_50_ = 27.45 ± 1.65 µM). Similarly, the remaining compounds showed good to moderate activity with IC_50_ values from 27.45 ± 1.65 µM to 251.74 ± 6.82 µM. The potency of compounds **6** and **25** possibly will be due to the presence of anthracene moiety and dimethoxy at 3 and 4 positions (*meta*, *para*) on the benzene ring, respectively. SAR suggested that the nature of substituent played a vital role. On the above results, these compounds deserve further research as a novel class of urease enzyme inhibitors.

## Figures and Tables

**Table 1:** Ar groups of synthesized *N′*-benzylidene-4-(t-Bu)benzohydrazide derivatives (**4**–**26**).

Compds.	Ar	Compds.	Ar	Compds.	Ar
**4**	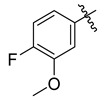	**12**	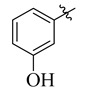	**20**	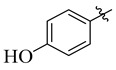
**5**	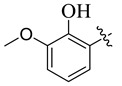	**13**	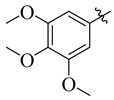	**21**	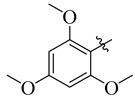
**6**	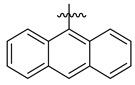	**14**	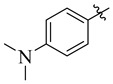	**22**	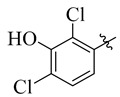
**7**	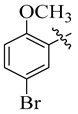	**15**	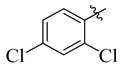	**23**	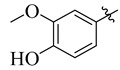
**8**	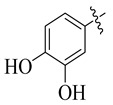	**16**	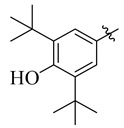	**24**	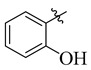
**9**	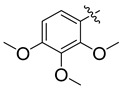	**17**	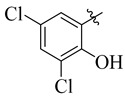	**25**	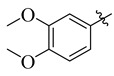
**10**	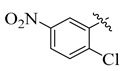	**18**	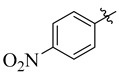	**26**	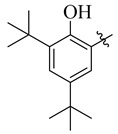
**11**	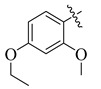	**19**	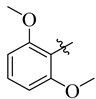	-	-

**Table 2:** Crystal and refinement data of complexes **5** and **14**.

Complex	5	14
Empirical formula	C_19_H_24_N_2_O_4_	C_20_H_25_N_3_O
Formula mass	344.40	323.43
Temperature (K)	170 K	170 K
Wavelength (Å)	0.71073	0.71073
Crystal system, space group	Monoclinic, *Cc*	Monoclinic, *P*2_1_/*n*
Unit cell dimensions (Å)	*a* = 7.1944 (14) Å, b = 41.282 (8) Å, *c* = 6.4595 (13) Å, β = 104.83 (3)°	*a* = 12.408 (3) Å, *b* = 8.5968 (17) Å, *c* = 35.223 (7) Å
Z, Volume (Å^3^)	1854.6 (7)	3757.2 (14)
Crystal size (mm)	0.48 × 0.47 × 0.10	0.48 × 0.17 × 0.12
Calculated density (Mg m^−3^)	1.233	1.144
Absorption coefficient (mm^−1^ )	0.09	0.07
F(000)	736	1392
Θ range for data collection	6.5–58.9°	6.2–59.1°
Limiting indices	*h* = −9→9, *k* = −56→47, *l* = −8→8	*h* = −13→13, *k* = −9→9, *l* = −38→38
Measured reflections	10166	20065
Independent reflections	4887	2509
Reflections with I > 2σ(I)	3476	1888
R_int_	0.105	0.247
Refinement method	Full-matrix least-squares on F^2^	Full-matrix least-squares on F^2^
Data/restraints/ parameters	4887/246/3	2509/0/230
*R*[*F*^2^ > 2σ(*F*^2^)]	0.072	0.079

**Table 3:** Selected bond lengths (Å) and bond angles (°) of 4-(t-Bu)-*N′*-(2-hydroxy-3-methoxybenzylidene)benzohydrazide (**5**).

**Moiety**	**Bond Length, Å**	**Moiety**	**Bond Length, Å**
O1—C1	1.356 (4)	N1—C7	1.284 (5)
O2—C2	1.368 (4)	N1—N2	1.364 (4)
O2—C19	1.413 (5)	N2—C8	1.349 (5)
O3—C8	1.230 (5)	C8—C9	1.492 (6)
C6—C7	1.451 (5)	C12—C15	1.528 (6)
**Moiety**	**Bond Angle, °**	**Moiety**	**Bond Angle, °**
C2—O2—C19	116.6 (3)	N1—C7—C6	119.9 (3)
C7—N1—N2	117.0 (3)	O3—C8—N2	121.6 (4)
C8—N2—N1	118.4 (3)	O3—C8—C9	122.1 (4)
O1—C1—C6	123.1 (3)	N2—C8—C9	116.3 (3)
O1—C1—C2	117.4 (3)	C14—C9—C10	117.4 (4)
O2—C2—C3	125.2 (4)	C14—C9—C10	117.4 (4)
O2—C2—C1	114.1 (3)	C1—C6—C7	121.2 (3)

**Table 4:** Hydrogen-bond geometry with selected bond lengths (Å) and bond angles (°).

*D*—H···*A*	*d(D—H)*	d(H···*A*)	*d(D···A)*	*∠* *(D—H···A)*
O1—H1*O*···N1	0.87 (6)	1.84 (6)	2.581 (4)	142 (6)
N2—H2*N*···O4 ^i^	0.85 (7)	2.00 (7)	2.808 (4)	160 (6)
C7—H7···O4 ^i^	0.95	2.47	3.250 (5)	139
C19—H19*A*···O2 ^ii^	0.98	2.57	3.259 (6)	127
O4—H4*O*···O3	0.81 (5)	1.89 (5)	2.696 (4)	174 (6)
O4—H4*P*···O1 ^iii^	0.84 (5)	2.19 (6)	2.967 (4)	154 (6)
O4—H4*P*···O2 ^iii^	0.84 (5)	2.42 (6)	3.025 (4)	130 (6)

Symmetry codes: (i) *x* + 1, *y*, *z*; (ii) *x*, −*y* + 1, *z*−1/2; (iii) *x*, *y*, *z* + 1.

**Table 5:** Selected bond lengths (Å) and bond angles (°) of compound **14**.

**Moiety**	**Bond Length, Å**	**Moiety**	**Bond Length, Å**
N1—C3	1.386 (4)	N2—N3	1.395 (3)
N1—C2	1.434 (5)	N3—C10	1.332 (4)
N1—C1	1.437 (6)	N3—H1N3	0.83 (3)
N2—C9	1.260 (4)	O1—C10	1.237 (3)
**Moiety**	**Bond Angle, °**	**Moiety**	**Bond Angle, °**
C3—N1—C2	119.2 (4)	N1—C3—C4	121.9 (3)
C3—N1—C1	119.9 (3)	C8—C3—N1	120.4 (3)
C2—N1—C1	118.3 (3)	N2—C9—C6	122.9 (3)
C9—N2—N3	114.4 (2)	N2—C9—H9	123.3 (19)
C10—N3—N2	120.7 (2)	O1—C10—N3	122.9 (2)
C10—N3—H1N3	127 (2)	O1—C10—C11	120.7 (2)
N2—N3—H1N3	113 (2)	N3—C10—C11	116.4 (2)
N1—C1—H1A	109.5	N1—C1—H1B	109.5

**Table 6:** Hydrogen-bond geometry with bond lengths (Å) and bond angles (°).

*D*—H···*A*	d(*D*—H)	d(H···*A*)	d(*D*···*A*)	∠(*D*—H···*A*)
N3—H1*N*3···O1 ^i^	0.83 (3)	2.12 (3)	2.939 (3)	169 (3)
C9—H9···O1 ^i^	0.90 (3)	2.57 (3)	3.322 (4)	142 (2)

Symmetry code: (i) −*x* + 3/2, *y* − 1/2, *z*.

**Table 7:** In vitro urease inhibitory activity of synthesized *N′*-benzylidene-4-(t-Bu)benzohydrazide derivatives (**4**–**26**).

Compounds	IC_50_ ± SEM (µM)	Compounds	IC_50_ ± SEM (µM)
**4**	88.75 ± 7.71	**16**	79.01 ± 7.53
**5**	56.57 ± 3.18	**17**	27.45 ± 1.65
**6**	13.33 ± 0.58	**18**	66.14 ± 1.79
**7**	64.50 ± 3.97	**19**	67.48 ± 4.16
**8**	80.01 ± 7.43	**20**	51.41 ± 11.50
**9**	63.50 ± 3.97	**21**	71.27 ± 2.47
**10**	63.54 ± 3.97	**22**	38.57 ± 11.54
**11**	63.52 ± 2.97	**23**	251.74 ± 6.82
**12**	39.57 ± 11.54	**24**	37.47 ± 12.54
**13**	80.93 ± 7.43	**25**	13.42 ± 0.33
**14**	81.21 ± 7.4	**26**	49.47 ± 12.74
**15**	82.21 ± 7.43	**Thiourea**	21.14 ± 0.42

SEM is the standard error of the mean, thiourea = standard for urease inhibition activity.

**Table 8:** Docking scores (S) and Interactions Detail of the selected active compounds (**9**–**11**, **16**, **18**, **19**, **22**, **24**, and **25**).

Compds.	Docking Score	Interactions Details				
		Ligand	Receptor	Interaction	Distance (Å)	E (kcal/mol)
**9**	−3.05	C23	5-ring-HIS324	π-H	3.83	−0.8
		6-ring	NZ-LYS169	π-cation	4.01	−1.4
**10**	−5.18	N9	SG-CYS322	HBD	3.13	−3.5
		O24	NI-NI798	metal	1.63	−2.0
		6-ring	CB-MET367	π-H	4.38	−0.6
**11**	−4.14	6-ring	CD-LYS169	π-H	4.32	−0.8
**16**	−4.71	CL9	NE2-HIS137	HBD	3.65	−1.3
		6-ring	CD-LYS169	π-H	4.14	−0.9
**18**	−12.09	C3	OD2-ASP363	HBD	3.30	−1.7
		O23	NI-NI798	metal	1.92	−2.9
		O24	NI-NI799	metal	1.89	−2.4
**19**	−3.03	N8	SG-CYS322	HBD	4.09	−0.8
**22**	−2.81	CL7	OD2-ASP363	HBD	2.73	1.2
**24**	−8.65	O7	OD2-ASP363	HBD	2.45	0.3
		6-ring	CD-LYS169	π-H	4.11	−0.7
**25**	−10.07	N8	O-CYS322	HBD	2.68	−2.1
		O24	NI-NI 798	metal	1.79	−1.8
		6-ring	CD-LYS169	π-H	4.23	−0.8
		6-ring	NE2-HIS222	π-H	4.18	−0.6

## Data Availability

The data presented in this study are available from the authors on reasonable request.

## Sample Availability

Samples of the compounds are available from the authors.
